# Supplementary material for: Interneurons and oligodendrocyte progenitors form a structured synaptic network in the developing neocortex
Source: eLife. 2015 Apr 22;4:e06953. doi: 10.7554/eLife.06953 (PMC4432226; doi:10.7554/eLife.06953)
Supplement: Supplementary file 1. — Synaptic coupling probabilities. DOI: http://dx.doi.org/10.7554/eLife.06953.020 [file elife06953s001.pdf]

Synaptic coupling probabilities

Christophe Pouzat

<2014-05-12 lun.>

1 Introduction

We have the following data set distinguishing between two interneurons types: `FS` (fast spiking) and `NFS` (not fast spiking), and for each type the oberved number of connection to a given post-synpatic cell type:

	`FS`	`NFS`	`Total`
Nb. (K)	35	112	147
Connected (Y)	15	23	48

We want to know if the difference between the observed connection probabilities:

- $\hat{p}_{\mathrm{FS}} = 15/35 \approx 0.43$
- $\hat{p}_{\mathrm{NFS}} = 23/112 \approx 0.21$

are "real" or could have arise by luck.

2 First approach

We model each of the two data sets (`FS` and `NFS`) as observations from two binomial distributions:

- $Y_{\mathrm{FS}} \sim \mathcal{B}\rangle\backslash(K_{\mathrm{FS}} = 35, p_{\mathrm{FS}})$,
- $Y_{\mathrm{NFS}} \sim \mathcal{B}\rangle\backslash(K_{\mathrm{NFS}} = 112, p_{\mathrm{NFS}})$.

We would then have:

- $\mathrm{E}Y_{\mathrm{T}} = K_{\mathrm{T}}\, p_{\mathrm{T}}$,
- $\mathrm{Var}Y_{\mathrm{T}} = K_{\mathrm{T}}\, p_{\mathrm{T}}\, (1 - p_{\mathrm{T}})$,

where T takes one of the two, `FS` and `NFS`, values.

Our estimates $\hat{p}_{\mathrm{T}} = Y_{\mathrm{T}}/K_{\mathrm{T}}$ would then have the following expected values and variance:

- $\mathrm{E}\hat{p}_{\mathrm{T}} = p_{\mathrm{T}}$,
- $\mathrm{Var}\hat{p}_{\mathrm{T}} = p_{\mathrm{T}}\,(1-p_{\mathrm{T}})/K_{\mathrm{T}} \approx \hat{p}_{\mathrm{T}}\,(1-\hat{p}_{\mathrm{T}})/K_{\mathrm{T}}$.

It is tempting from this point to use the normal approximation to build (Wald) confidence intervals obtained by inverting a Wald test. But these confidence intervals aren't that good as show by Brown, Cai and DasGupta [1]. Among the better alternatives recommended in the latter publication we can use the *arcsine interval* based on the variance stabilization transformation of Anscombe [2]. The latter defines first the following proportion estimate:

$$\check{p}_{\mathrm{T}} = \frac{Y_{\mathrm{T}} + 3/8}{K_{\mathrm{T}} + 3/4}\,.$$

A confidence interval of level α is then obtained with:

$$\left[\sin^2\left(\arcsin\left(\sqrt{\check{p}}\right) - \frac{\kappa}{2\sqrt{K_{\mathrm{T}}}}\right), \sin^2\left(\arcsin\left(\sqrt{\check{p}}\right) + \frac{\kappa}{2\sqrt{K_{\mathrm{T}}}}\right)\right]\,,$$

where

$$\kappa = \Phi^{-1}(1-\alpha/2)$$

and Φ is the cumulative distribution function of the standard normal distribution (mean 0 and variance 1).

We are going to compute 90% confidence intervals for each of the two samples. The probability (α) for the true connection probability to be out of the interval will then be 0.1 (1-0.9). If we then assume that the two samples have **the same true connection probability** and if the two confidence intervals do not overlap, we can reject the null hypothesis (**same connection probability**) with a 0.99 (0.1 x 0.1) confidence level.

2.1 Using `Python`

We use `Python 3` even if the following commands should give the same results with `Python 2`. So we start the `Python` shell and import the necessery modules:

[1]Lawrence D. Brown, T. Tony Cai and Anirban DasGupta (2001) Interval Estimation for a Binomial Proportion. *Statistical Science* **16**: 101-133.

[2]F. J. Anscombe (1948) The Transformation of Poisson, Binomial and Negative-Binomial. *Biometrika* **35**: 246-254.

```
import numpy as np
from scipy.stats import binom
from scipy.stats import norm
```

We then define the required variables:

```
y_FS = 15
K_FS = 35
y_NFS = 23
K_NFS = 112
```

We get the $\check{p}$:

```
p_check_FS = (y_FS + 3./8.)/(K_FS + 3./4.)
p_check_NFS = (y_NFS + 3./8.)/(K_NFS + 3./4.)
```

We get κ:

```
kappa = norm.ppf(0.95)
```

The 90% confidence interval for $\check{p}_{\mathrm{FS}}$ is therefore:

```
CI_90_FS = (np.sin(np.arcsin(np.sqrt(p_check_FS)) - kappa/np.sqrt(K_FS)/2.)**2,
            np.sin(np.arcsin(np.sqrt(p_check_FS)) + kappa/np.sqrt(K_FS)/2.)**2)
CI_90_FS
```

0.2968728130643559	0.5686379957493932

And the 90% confidence interval for $\check{p}_{\mathrm{NFS}}$ is:

```
CI_90_NFS = (np.sin(np.arcsin(np.sqrt(p_check_NFS)) - kappa/np.sqrt(K_NFS)/2.)**2,
             np.sin(np.arcsin(np.sqrt(p_check_NFS)) + kappa/np.sqrt(K_NFS)/2.)**2)
CI_90_NFS
```

0.14809191938104865	0.27359824116324444

The two intervals **do not overlap** and we can reject the null with a 99% confidence.

3 Second approach

The recommend way to built the confidence intervals in Brown, Cai and DasGupta [1] is in fact the use of the *Wilson interval* defined by:

$$\left[\frac{Y_\mathrm{T}+\kappa^2/2}{K_\mathrm{T}+\kappa^2}-\frac{\kappa\sqrt{K_\mathrm{T}}}{K_\mathrm{T}+\kappa^2}\sqrt{\hat{p}(1-\hat{p})+\frac{\kappa^2}{4K_\mathrm{T}}},\frac{Y_\mathrm{T}+\kappa^2/2}{K_\mathrm{T}+\kappa^2}+\frac{\kappa\sqrt{K_\mathrm{T}}}{K_\mathrm{T}+\kappa^2}\sqrt{\hat{p}(1-\hat{p})+\frac{\kappa^2}{4K_\mathrm{T}}}\right].$$

3.1 Using Python

The 90% confidence interval for $\check{p}_{\mathrm{FS}}$ is therefore:

```
p_hat_FS = y_FS/K_FS
CI_Wilson_90_FS = ((y_FS+kappa**2/2)/(K_FS+kappa**2) -
                   kappa*np.sqrt(K_FS)/(K_FS+kappa**2)*np.sqrt(p_hat_FS*(1-p_hat_FS)+
                   kappa**2/K_FS/4.),
                  (y_FS+kappa**2/2)/(K_FS+kappa**2) +
                   kappa*np.sqrt(K_FS)/(K_FS+kappa**2)*np.sqrt(p_hat_FS*(1-p_hat_FS)+
                   kappa**2/K_FS/4.))
CI_Wilson_90_FS
```

0.30103625941541223   0.5663572445109468

And the 90% confidence interval for $\check{p}_{\mathrm{FS}}$ is:

```
p_hat_NFS = y_NFS/K_NFS
CI_Wilson_90_NFS = ((y_NFS+kappa**2/2)/(K_NFS+kappa**2) -
                    kappa*np.sqrt(K_NFS)/(K_NFS+kappa**2)*
                    np.sqrt(p_hat_NFS*(1-p_hat_NFS)+
                    kappa**2/K_NFS/4.),
                   (y_NFS+kappa**2/2)/(K_NFS+kappa**2) +
                    kappa*np.sqrt(K_NFS)/(K_NFS+kappa**2)*
                    np.sqrt(p_hat_NFS*(1-p_hat_NFS)+
                    kappa**2/K_NFS/4.))
CI_Wilson_90_NFS
```

0.14987821338072027   0.27473547125420866

Again, no overlap.

4 Third approach

We consider the difference $\hat{p}_{\mathrm{FS}} - \hat{p}_{\mathrm{NFS}}$ and we estimate the cumulative distribution of this difference under the null hypothesis: $p_{\mathrm{FS}} = p_{\mathrm{NFS}} = p$. The estimate $\hat{p}$ of p is then:

$$\hat{p} = \frac{Y_{\mathrm{FS}} + Y_{\mathrm{NFS}}}{K_{\mathrm{FS}} + K_{\mathrm{NFS}}} \approx 0.26$$

We then simulate 10000 samples with 35 + 112 events each of them having the same probability $\hat{p}$ of being connected. For each simulated sample (n) we compute the difference $\hat{p}_{\mathrm{FS}}^{(n)} - \hat{p}_{\mathrm{NFS}}^{(n)}$. We then get the cumulative distribution function of these 10000 differences and we look at the probability of obtaining (under the null hypothesis) a difference equal to or larger than the one we actually observed.

4.1 Using `Python`

First in order to get reproducible simulations, we explicitely seed our (pseudo)random number generator:

```
np.random.seed(20061001)
```

We then do our simulations very simply with:

```
n_replicates = 10000
p_hat = (y_FS+y_NFS)/(K_FS+K_NFS)
res = binom.rvs(K_FS,p_hat,size=n_replicates)/K_FS-
      binom.rvs(K_NFS,p_hat,size=n_replicates)/K_NFS
```

The probabilty of obtaining a difference equal to or larger than the one we observed is then:

```
p_diff_obs=y_FS/K_FS-y_NFS/K_NFS
np.sum(res >= p_diff_obs)/n_replicates
```

```
0.0062
```

Again smaller than 0.01...

With `python 2`, one should use:

```
p_diff_obs=float(y_FS/K_FS-y_NFS/K_NFS)
```

to compute `p_diff_obs`.
